# A Century of Post-Traumatic Appendicitis: A Comprehensive Review with an Illustrative Case

**DOI:** 10.3390/pediatric18030079

**Published:** 2026-06-10

**Authors:** Mattia Pasquinucci, Irene Marangoni, Veronica Battistella, Maria E. Pinto, Alessandra Pasinato, Fabio S. Chiarenza, Davide Meneghesso

**Affiliations:** 1 Department of Pediatrics, AULSS 7 Pedemontana-San Bassiano Hospital, 36061 Bassano del Grappa, Italy; mariaelisabettapinto@gmail.com (M.E.P.);; 2 Department of Pediatrics, University of Padua, 35100 Padua, Italy; 3 Department of Pediatric Surgery, AULSS 8 Berica-San Bortolo Hospital, 36100 Vicenza, Italy

**Keywords:** acute appendicitis, blunt abdominal trauma, pediatric appendicitis, conservative management, post-traumatic appendicitis

## Abstract

**Background and Clinical Significance:** Acute appendicitis following blunt abdominal trauma is a rare and historically debated clinical entity. We present a century-spanning descriptive review of 106 cases of post-traumatic appendicitis, embedded with an illustrative pediatric case initially managed conservatively. **Methods:** A comprehensive literature review was conducted following PRISMA guidelines across PubMed/MEDLINE, Web of Science, and Google Scholar, encompassing a 100-year period (1925–2025). Clinical variables, trauma mechanisms, and outcomes were extracted and statistically analyzed by age cohort (Pediatric ≤ 18 vs. Adult > 18) and historical medical era. **Results:** A total of 106 cases were analyzed. High-energy trauma predominated in adults compared to the pediatric cohort (48.8% vs. 18.5%, *p* = 0.001). The overall complication rate was exceptionally high (66.0%), with no significant difference between pediatric and adult cohorts (61.5% vs. 73.2%, *p* = 0.293). An epoch-based analysis revealed a significant drop in perforation rates from the historical era (1925–1980) to the modern era (2001–2025) (51.7% to 27.0%, *p* = 0.033) due to improved diagnostic timelines. Crucially, purely mechanical injuries such as complete appendiceal auto-amputation remained a constant signature of blunt trauma across the century (11.5% overall rate). **Conclusions:** Our synthesis of historical cases suggests that post-traumatic appendicitis might be a relevant clinical entity where trauma mechanics appear to play a significant role in injury severity, irrespective of patient age. While conservative management could be feasible and safe in the acute setting of uncomplicated cases, we hypothesize that the initial kinetic impact might cause subtle structural changes or alter local appendiceal dynamics, potentially predisposing the organ to recurrent inflammation, warranting close follow-up or elective surgery.

## 1. Introduction and Clinical Significance

Acute appendicitis is one of the most common reasons for emergency department visits related to surgical conditions, particularly in the paediatric population. Its incidence is approximately 100 per 100,000 children [1]. The diagnosis of this condition is generally clinical in nature and can be confirmed through a variety of investigative methods, including blood tests and imaging techniques. Abdominal ultrasound is the modality of choice for initial assessment. The utilisation of computed tomography (CT) and magnetic resonance imaging (MRI) of the abdomen is restricted to a select group of patients. The primary causes of appendicitis are well-documented and widely recognised. However, among the less common etiological factors, there is one whose existence is still subject to debate and controversy: post-traumatic appendicitis (PTA). The first documented account of this phenomenon was Murphy in 1982 [2]. As posited by Fowler et al. [3], the diagnosis of PTA is made on the basis of four criteria: the absence of a prior history of abdominal attacks; the presence of direct or severe indirect abdominal trauma; the onset of symptoms in close temporal proximity to the trauma; and the progression of symptoms that ultimately result in the diagnosis and treatment of appendicitis. However, some authors argue, particularly given the high frequency of abdominal trauma in paediatric patients, that the occurrence of appendicitis in this cohort may be purely coincidental [4]. In order to explore this phenomenon, the present paper is structured as a systematic descriptive review of published cases with an embedded illustrative case. The clinical course of a paediatric patient with PTA managed conservatively is presented first. Subsequently, a century (1925–2025) of published literature was synthesised in order to define the descriptive clinicopathological profile of PTA. The objective of this study is not to generate robust epidemiological evidence, but rather to encourage paediatric colleagues to refrain from underestimating the potentially severe consequences of traumatic events. While the study’s primary focus is on the paediatric population, a comparative adult cohort was deliberately included in the comprehensive review. This approach provides a vital, necessary epidemiological baseline, essential for investigating whether the thinner abdominal wall and different baseline mechanisms of injury in children result in distinct clinicopathological profiles compared to adults.

## 2. Case Presentation

**Figure 1:**
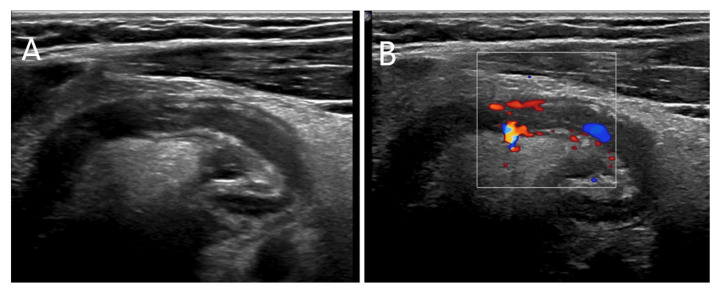
(**A**) shows signs of appendicitis (thickening of the appendix, increased echogenicity, and double-wall sign), while (**B**) shows coloroppler signal of the appendicular walls.

A 10-year-old male patient presented to our paediatric emergency department due to the onset of acute right iliac fossa pain that had begun within the previous few hours, without other associated symptoms. The patient denied fever, vomiting, diarrhea, nausea, peritoneal signs, or any prodromal symptoms in the preceding days. The only significant incident in his medical history was a direct trauma to the right iliac fossa while playing hockey. The collision between the boy and an opponent’s hockey stick was the result of a physical interaction between the two parties. Approximately 3.5 h following the incident, the patient began to experience a progressive exacerbation of abdominal pain. Following the preliminary paediatric examination, tenderness was observed in the abdominal region, accompanied by localised pain and muscle resistance upon palpation of the right iliac fossa. This finding was accompanied by a positive McBurney’s sign. The absence of bruising or significant signs of trauma was noted. The patient’s body temperature was recorded at approximately 37.4 °C (99.3 °F). The remaining physical examination yielded no significant findings. In order to exclude the possibility of solid organ injury resulting from blunt abdominal trauma, a urine test was performed (which was negative for hematuria), along with an abdominal ultrasound, which clearly demonstrated signs of appendicular inflammation. Specifically, there was thickening of the appendix (transverse diameter of 8 mm), increased echogenicity, and wall thickening with a double-wall sign, without free fluid or mesenteric fat stranding, and no reactive-looking abdominal lymphadenopathy (Figure 1A,B).

Laboratory tests showed elevated inflammatory markers (C-reactive protein 46.7 g/L, normal value <5 g/L), yet a normal blood count was observed (Table 1).

In light of the suspicion of PTA, the patient was admitted for clinical observation and initiated on antibiotic therapy (ampicillin/sulbactam with intravenous administration). Following a multidisciplinary consultation involving paediatricians, surgeons, and radiologists, a conservative approach was chosen due to the patient’s overall stable clinical condition.

Subsequent blood tests on the following day revealed a marginal increase in C-reactive protein, with no other substantial alterations. The patient demonstrated a swift clinical recovery. The abdominal pain resolved after approximately three days of antibiotic therapy. A subsequent examination, conducted one week after the onset of symptoms, showed a complete resolution of both laboratory and ultrasound findings. The patient was discharged in satisfactory general condition, with the recommendation that they abstain from participation in contact sports for a period of two weeks. There was no recurrence of symptoms during the following six months. Subsequently, the patient returned to the emergency department, manifesting symptoms that were substantially analogous to those experienced during the initial episode. These symptoms were characterised by right iliac fossa pain in the absence of vomiting, nausea, peritoneal irritation, or a significant febrile response. As demonstrated in Table 2 and Table 3, both the Acute Inflammatory Response (AIR) and Alvarado scores consistently indicated a low probability of acute appendicitis [5,6,7,8].

The Pediatric Appendicitis Score (PAS) confirmed the low probability of appendicitis (Table 4) [9,10].

Laboratory investigations were notable solely for a mildly elevated C-reactive protein level of 21.4 g/L (Table 5).

In view of the persistent nature of the symptoms and the second ultrasound examination once again demonstrating findings suggestive of appendicitis, the patient underwent surgical appendectomy. Histopathological analysis confirmed the diagnosis of catarrhal appendicitis. Postoperative recovery was uneventful, and the patient was discharged home in a stable condition. At the 12-month follow-up stage, no symptoms were observed.

## 3. Materials and Methods

### 3.1. Search Strategy and Selection Criteria

A systematic review of the literature was conducted in accordance with the Preferred Reporting Items for Systematic Reviews and Meta-Analyses (PRISMA) guidelines. A comprehensive literature search was performed across PubMed/MEDLINE, Web of Science, and Google Scholar databases, covering a 100-year period from January 1925 to December 2025. The search strategy employed a combination of the following keywords and Medical Subject Headings (MeSH) terms: “post-traumatic appendicitis” OR “posttraumatic appendicitis” OR “appendicitis injury” OR “blunt abdominal trauma” OR “BAT” OR “traumatic appendicitis”.

### 3.2. Inclusion and Exclusion Criteria

In order to ensure the rigorous selection of relevant cases, articles were included if they met the following criteria: (1) original case reports or case series describing acute appendicitis following blunt abdominal trauma; (2) peer-reviewed publications. Articles were excluded if they were: (1) duplicate publications; (2) articles lacking clear temporal correlation between trauma and symptom onset; or (3) off-topic studies focusing on penetrating trauma or incidental appendectomies.

In order to minimise the impact of language bias and ensure the most comprehensive review possible, non-English articles were not excluded a priori. The translation of these articles into English was facilitated by a large language model (Gemini, Version 2.5 Pro, Google LLC). In order to mitigate potential errors in translation or misinterpretations of historical medical terminology, all extracted clinical variables (e.g., surgical findings, timeline of symptom onset) were independently cross-checked by the authors for clinical coherence and contextual accuracy.

### 3.3. Data Extraction

**Figure 2:**
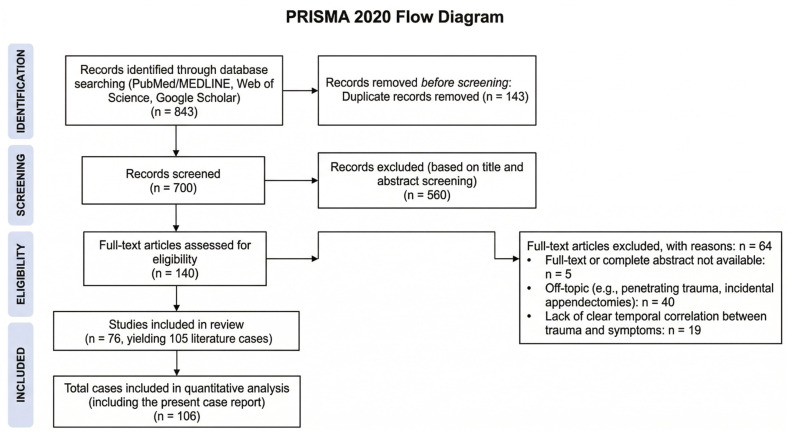
PRISMA flow diagram of the study selection process.

In the initial phase of the research, a total of 843 articles were retrieved from the database. Following the removal of duplicates and preliminary title/abstract screening, the full-text articles were subjected to a rigorous evaluation to ascertain their eligibility. In the final analysis, 76 articles met the inclusion criteria. Including our present case, a total of 106 well-documented cases of post-traumatic appendicitis were included in the final quantitative analysis (Figure 2).

Data extracted included patient demographics (age, sex), mechanism of injury (categorized as high-energy or low-energy trauma), time to symptom onset, imaging modalities, presence of appendicolith, operative versus conservative management, and specific clinical outcomes (e.g., uncomplicated, perforation, peritonitis, auto-amputation).

### 3.4. Statistical Analysis

Patients were stratified into two primary cohorts based on age: Pediatric (≤18 years) and Adult (>18 years). In order to evaluate the impact of evolving medical diagnostics, cases were further divided into three historical eras: Historical (1925–1980), Transition (1981–2000), and Modern (2001–2025). Categorical variables were reported as frequencies and percentages. Continuous laboratory variables were reported as medians due to non-normal distribution. Comparisons between groups for categorical variables (e.g., complication rates, mechanisms of injury) were performed using Fisher’s Exact Test. A two-tailed *p*-value < 0.05 was considered statistically significant.

## 4. Results of the Comprehensive Literature Review

### 4.1. Baseline and Trauma Characteristics

A total of 106 cases of PTA were identified and stratified into a paediatric cohort (≤18 years, N = 65) and an adult cohort (>18 years, N = 41). The prevalence of male subjects was observed to be significantly high in both cohorts, with an overall proportion of 77.4%, exhibiting no substantial variation across the study groups. A statistically significant difference in the mechanism of injury was identified: high-energy blunt trauma (e.g., motor vehicle collisions, seatbelt injuries) accounted for 48.8% of adult cases compared to only 18.5% in the paediatric cohort (*p* = 0.001). Conversely, paediatric PTA was predominantly associated with low-energy focal impacts such as bicycle falls or sports-related kicks. Appendicoliths were reported in approximately one-fifth of cases across both groups, showing no age-related predilection (*p* = 0.809, Table 6). A comprehensive, case-by-case breakdown of the clinical presentations, specific injury mechanisms, diagnostic modalities, and clinical outcomes for all 106 compiled patients is provided in Table A1.

### 4.2. Clinical Presentation and Diagnostic Findings

The prevalence of abdominal pain as a presenting symptom was found to be overwhelming, with a reported 99.1% of the population experiencing this symptom. Pediatric patients exhibited a tendency towards a more reactive systemic presentation, characterised by elevated rates of vomiting (44.6% vs. 25.7%, *p* = 0.079) and fever (32.8% vs. 17.1%, *p* = 0.147) when compared to adults. However, these differences did not attain strict statistical significance (Table 7).

Laboratory data, predominantly available for cases in the modern medical era, confirmed prominent leukocytosis and only mild elevated inflammatory markers across both cohorts (Table 8).

The diagnostic approach to post-traumatic appendicitis has undergone a profound shift alongside the evolution of emergency imaging. Prior to the year 2000, surgical intervention was the prevailing mode of diagnosis (90.5%, n = 38/42), as patients routinely underwent urgent exploratory laparotomy for blunt abdominal trauma, often without advanced pre-operative imaging. Conversely, in the modern era (2000–2025, n = 63), the diagnostic workflow became heavily reliant on non-invasive imaging. The present study found that computed tomography (CT) emerged as the predominant definitive modality (61.9%, n = 39/63) to confirm appendiceal injury and rule out solid organ lesions. Ultrasonography (US) was successfully utilised in 15.9% of cases (n = 10/63), primarily as a radiation-free first-line tool in children. Nevertheless, 22.2% of contemporary patients (n = 14/63) still required surgical exploration due to persistent symptoms or technical limitations of imaging (e.g., bowel gas, abdominal wall haematomas), underscoring that serial clinical examination remains the ultimate safety net.

### 4.3. Complication Patterns Across a Century of Practice

The overall complication rate for post-traumatic appendicitis was found to be remarkably high (66.0%), with no significant difference observed between paediatric (61.5%) and adult (73.2%) cohorts (*p* = 0.293). This finding suggests that age is not a determining factor in the severity of outcomes. In order to address the century-long scope of this review and mitigate historical diagnostic bias, outcomes were stratified by medical eras. A dramatic, statistically significant reduction in overall complications was observed, dropping from 93.1% in the Historical Era (1925–1980) to 53.9% in the Modern Era (2001–2025) (*p* = 0.0001). Specifically, purely biologically driven complications such as perforation nearly halved (51.7% to 27.0%, *p* = 0.033), and gangrene showed a similar drastic decline. Conversely, mechanically driven complications—such as auto-amputation—remained a constant signature of blunt trauma across the century (11.5% overall rate), unaffected by modern diagnostic advancements (Table 9).

## 5. Discussion

The association between blunt abdominal trauma and acute appendicitis has been described on multiple occasions in the literature [11,12,13], although its existence remains a topic of debate. A recent study by Habachi et al. reviewed 51 cases of PTA reported over the past 33 years [14]. To expand upon this, our review encompasses 106 cases over a full century (1925–2025), providing the most comprehensive historical and descriptive synthesis of published cases to date (Table A1). The temporal relationship between the triggering event and the onset of appendicular pain, which occurs in the absence of prior symptoms, appears to be consistent. The triggering event may be categorised as one of several potential causes, including, but not limited to, seatbelt injuries, road traffic accidents, assaults, and falls. It is also possible that more rare occurrences, such as impacts from footballs or kicks, may be associated with the onset of appendicular pain. The patient in question shares common characteristics with the aforementioned cases, as his abdominal pain developed approximately three hours after the trauma. While it is physiologically challenging for *de novo* bacterial inflammation to develop to the point of wall thickening in such a short timeframe, the trauma probably acts as an immediate precipitating factor. It is noteworthy that the clinical likelihood of appendicitis in our case appeared low, as indicated by the Alvarado, AIR, and PAS scales. Despite this, the ultrasound findings and their subsequent normalization left little doubt regarding the diagnosis, which was later confirmed by histopathology following the patient’s second presentation.

We observed a constant 11.5% rate of purely mechanical complications—such as complete appendiceal transection or auto-amputation—across all medical eras. This constant baseline of structural shearing may support the hypothesis that direct kinetic energy transfer might severely injure the appendix, independent of pre-existing inflammation or diagnostic timing. In less disruptive injuries, several Authors suggest that direct trauma causes secondary edema or hematoma, leading to stenosis of the appendiceal lumen and initiating a non-bacterial inflammatory process [15,16]. In this particular instance, the patient’s appendix was located directly beneath the oblique muscle, partially anterior to the cecal wall. This anatomical feature has been hypothesised to provide a possible explanation for the development of appendicitis even in cases where mild trauma has been sustained. This is of particular relevance in paediatric patients. Their propensity to develop post-traumatic appendicitis extends beyond the mere presence of a thinner abdominal wall. It is important to note that children possess a unique biomechanical profile, which amplifies energy transmission to the right iliac fossa. Specifically, the shallower configuration of the pelvis relative to the cecum results in diminished protection by the bony ring. Furthermore, a highly compliant thoracic cage deforms rather than fractures, transmitting compressive forces directly to underlying viscera. This is further exacerbated by a deficiency in developed musculature and subcutaneous fat, which significantly reduces shock-absorbing capacity. In conclusion, the structurally short and immature paediatric greater omentum is inadequate in adequately cushioning impacts or containing early localised inflammation. The combined anatomical factors underpin the phenomenon that even mild blunt trauma can generate sufficient shearing forces to severely injure the appendix in children.

A subsequent exploratory comparative analysis revealed no significant difference in overall complication rates between paediatric (61.5%) and adult (73.2%) cohorts (*p* = 0.293). While adults are significantly more exposed to high-energy trauma like motor vehicle collisions (48.8% vs. 18.5%, *p* = 0.001), the vulnerability of the appendiceal region to focal kinetic impact remains high across all age demographics. The elevated C-reactive protein, in the absence of changes in procalcitonin or leukocyte count, in this patient suggests a purely inflammatory process driven by post-traumatic stress [17], without concomitant infection. More severe or delayed cases may be linked to secondary bacterial infection, potentially driven by fecal stasis or appendicoliths (present in over 20% of our pooled cases). These factors could facilitate bacterial overgrowth once traumatic oedema narrows the lumen.

From a management perspective, this case offers a novel insight. To the best of our knowledge, the extant literature on PTA has focused predominantly on surgical outcomes. Our preliminary conservative approach led to a symptom-free interval of several months. However, following recurrence, surgical intervention was undertaken. A high perforation rate has been documented in the literature; however, epoch-based analysis (Table 9) reveals a significant historical diagnostic bias. In the era preceding 1980, the overall complication rate was found to be 93.1%, with a perforation rate of 51.7%. In the modern era (2001–2025), the prevalence of perforations has decreased by almost 50%. This decline does not suggest a biological reduction in the severity of the disease; rather, it might be a consequence of the emergence of advanced imaging techniques (e.g., CT, FAST ultrasound) that facilitate the incidental detection of an edematous appendix prior to its progression to gangrene. Historically, a ‘wait-and-see’ approach led to delayed laparotomies performed only when frank peritonitis was evident.

Despite modern early detection, the recurrence in our conservatively managed patient raises an important question regarding long-term efficacy. Our hypothesis is that the initial kinetic impact may have resulted in subtle structural changes or altered local appendiceal dynamics, which may have predisposed the organ to recurrent inflammation. However, the final histopathology revealed only catarrhal appendicitis, without any evidence of macroscopic scarring or strictures. Consequently, this mechanism remains speculative.

In view of the limited awareness surrounding the precise etiopathogenesis, it is important to raise the awareness of this condition among paediatric and adult emergency physicians. The concurrence of localized trauma to the right iliac fossa and the subsequent onset of appendicitis is rarely coincidental. Creating data registries and further studies will facilitate a more precise delineation of the role and safety of non-operative management in this unique nosological entity.

### Limitations to the Study

The present study is subject to several significant limitations. The primary concern with the systematic review is that it is based entirely on retrospective case reports and case series, which inherently carry a high risk of publication and selection bias. It was not possible to perform a meaningful formal quality appraisal of the included studies due to the historical nature of the data. Consequently, the statistical comparisons and *p*-values derived from pooling these historical cases must be interpreted with extreme caution; they are purely exploratory and hypothesis-generating rather than robust, confirmatory epidemiological evidence. Moreover, the absence of standardized demographic reporting in these historical sources precluded any meaningful epidemiological analysis of incidence or severity variations based on ethnicity, geographic region, or healthcare access. Finally, given that the historical approach to PTA was overwhelmingly surgical and retrospective case reports inherently lack long-term follow-up, there is currently insufficient evidence to accurately determine the long-term safety and recurrence rates of conservative management.

## 6. Conclusions

Despite the controversy surrounding its existence in the literature, it appears improbable that the concurrence of localized trauma to the right iliac fossa and the subsequent onset of appendicitis is purely coincidental. This condition may be significantly underdiagnosed, likely due to the lack of routine use of targeted ultrasound in cases of mild-to-moderate blunt abdominal trauma, especially when there is no haemodynamic instability, deterioration in general condition, or symptoms suggesting organ damage.

In light of the documented high incidence of surgical complications in reported cases, often presenting late to medical attention, clinicians are advised to maintain a high level of vigilance for the potential involvement of appendiceal structures in cases of mild-to-moderate abdominal trauma, particularly in instances of localised trauma and tenderness in the right iliac fossa. In this particular instance, an initial non-operative approach yielded prolonged clinical stability, thereby suggesting that conservative treatment may be a viable option for a select group of patients. This emphasises the necessity for additional research to be conducted into the safety and efficacy of conservative strategies within this particular context.

## Figures and Tables

**Table 1:** Laboratory findings showing a decline in white blood cell count and absolute neutrophil count by the second day of hospitalization. Notably, C-reactive protein (CRP) levels remained elevated (>45 g/L) despite the normalization of the leukocyte count, suggesting a persistent underlying inflammatory process or delayed biochemical resolution.

	At Admission	2nd Day	Normal Values
White blood cells (/mm^3^)	8640	5900	4500–13,500/mm^3^
Neutrophils (/mm^3^)	5790	2830	1500–8000/mm^3^
C-reactive protein (g/L)	46.7	49.8	<5 g/L

**Table 2:** Comparative scoring of symptoms and laboratory markers during two separate clinical presentations according to AIR Score [5,6]. Despite persistent right iliac fossa pain and elevated C-reactive protein, the total score remained low (2/12) due to the absence of high-grade fever, rebound tenderness, and significant leukocytosis.

Symptom/Sign—AIR Score	1st Episode	2nd Episode
Right iliac fossa pain	1/1	1/1
Vomiting	0/1	0/1
Rebound tenderness	0/3	0/3
Fever of 38.5 °C or more	0/1	0/1
Leukocytosis (>15,000/mm^3^)	0/2	0/2
Neutrophils (>85%)	0/2	0/2
C-reactive protein (>50 g/L)	1/2	1/2
Total	2/12	2/12

**Table 3:** Evaluation of inflammatory markers and clinical signs during the patient’s initial visit (first episode) and subsequent recurrence (second episode) according to Alvarado Score [7,8]. Scores of 3/10 and 2/10, respectively, indicate a “low probability” of acute appendicitis according to standardized diagnostic criteria.

Symptom/Sign—Alvarado Score	1st Episode	2nd Episode
Abdominal pain that migrates to the right iliac fossa	1/1	1/1
Anorexia (loss of appetite) or ketones in the urine	0/1	0/1
Nausea or vomiting	0/1	0/1
Tenderness in the right iliac fossa	1/2	1/2
Rebound tenderness	0/1	0/1
Fever of 37.3 °C or more	1/1	0/1
Leukocytosis (>10,000/mm^3^)	0/2	0/2
Neutrophils (>75%)	0/1	0/1
Total	3/10	2/10

**Table 4:** Evaluation of inflammatory markers and clinical signs during the patient’s initial visit (first episode) and subsequent recurrence (second episode). Pediatric Appendicitis Scores of 2/10 and 2/10 [9,10], respectively, indicate a “low probability” of acute appendicitis according to standardized diagnostic criteria.

Symptom/Sign—PAS Score	1st Episode	2nd Episode
Abdominal pain that migrates to the right iliac fossa	1/1	1/1
Anorexia	0/1	0/1
Nausea or vomiting	0/1	0/1
Tenderness in the right iliac fossa	1/2	1/2
Tenderness with coughing/percussion/hopping	0/2	0/2
Fever of 38 °C or more	0/1	0/1
Leukocytosis (>10,000/mm^3^)	0/1	0/1
Polymorphonuclear leukocytes (>75%)	0/1	0/1
Total	2/10	2/10

**Table 5:** The table shows biochemicals before and after surgery, with a slight decrease in C-reactive protein levels after appendicectomy.

	At Admission	At Discharge	Normal Values
White blood cells (/mm^3^)	5480	5150	4500–13,500/mm^3^
Neutrophils (/mm^3^)	2220	2660	1500–8000/mm^3^
C-reactive protein (g/L)	21.4	11.2	<5 g/L

**Table 6:** Baseline demographic, clinical, and trauma-related characteristics of the overall study population, stratified by pediatric and adult cohorts.

Variable	Overall (N = 106)	Pediatrics (N = 65)	Adults (N = 41)	*p*-Value
Sex (Male), n (%) [95% CI]	82 (77.4) [69.4–85.4]	50 (76.9) [66.7–87.1]	32 (78.0) [65.3–90.7]	1.00
High-Energy Trauma, n (%) [95% CI]	32 (30.2) [21.5–38.9]	12 (18.5) [9.1–27.9]	20 (48.8) [33.5–64.1]	0.001
Low-Energy Trauma, n (%) [95% CI]	74 (69.8) [61.1–78.5]	53 (81.5) [72.1–90.9]	21 (51.2) [35.9–66.5]	0.001
Primary Diagnosis Modality, n (%) [95% CI]				
- Computed Tomography (CT)	40 (37.7) [28.5–46.9]	22 (33.8) [22.3–45.3]	18 (43.9) [28.7–59.1]	0.312
- Ultrasound (US)	11 (10.4) [4.6–16.2]	9 (13.8) [5.4–22.2]	2 (4.9) [0.0–11.5]	0.197
- Surgical	52 (49.1) [39.6–58.6]	31 (47.7) [35.6–59.8]	21 (51.2) [35.9–66.5]	0.842
Presence of Appendicolith, n (%) [95% CI]	23/102 (22.5) [14.4–30.6]	15/62 (24.2) [13.5–34.9]	8/40 (20.0) [7.6–32.4]	0.809
Overall Complications, n (%) [95% CI]	70 (66.0) [57.0–75.0]	40 (61.5) [49.7–73.3]	30 (73.2) [59.7–86.7]	0.293
Need of Surgery, n (%) [95% CI]	105 (99.1) [97.3–100.0]	64 (98.5) [95.5–100.0]	41 (100.0) [100.0–100.0]	1.00

High-energy trauma includes motor vehicle, motorcycle, and seatbelt-related collisions; low-energy trauma includes falls, bicycle accidents, and sports-related kicks.

**Table 7:** Frequencies of reported clinical symptoms at initial presentation.

Variable	Overall	Pediatrics	Adults	*p*-Value
Abdominal Pain, n (%) [95% CI]	105/106 (99.1) [97.3–100.0]	65/65 (100) [100.0–100.0]	40/41 (97.6) [92.9–100.0]	0.386
Vomiting, n (%) [95% CI]	34/91 (37.4) [27.5–47.3]	25/56 (44.6) [31.6–57.6]	9/35 (25.7) [11.2–40.2]	0.079
Fever, n (%) [95% CI]	25/93 (26.9) [17.9–35.9]	19/58 (32.8) [20.7–44.9]	6/35 (17.1) [4.6–29.6]	0.147

**Table 8:** Median laboratory values recorded upon admission. Data predominantly reflect cases from the modern medical era.

Variable	Overall	Pediatrics	Adults	N Total
WBC (cells/μL)	14,800	16,050	13,750	40
Neutrophils (cells/μL)	11,500	10,000	14,250	11
C-Reactive Protein (mg/dL)	3.52	2.28	4.75	10

**Table 9:** Evolution of specific appendiceal complications and outcomes across three distinct historical medical eras.

Complication Type	Historical Era (N = 29) (1925–1980)	Transition Era (N = 13) (1981–2000)	Modern Era (N = 63) (2001–2025)	*p*-Value
Uncomplicated, n (%) [95% CI]	2 (6.9) [0.0–16.1]	3 (23.1) [0.2–46.0]	29 (46.0) [33.7–58.3]	<0.001
Perforation, n (%) [95% CI]	15 (51.7) [33.5–69.9]	6 (46.2) [19.1–73.3]	17 (27.0) [16.0–38.0]	0.033
Peritonitis, n (%) [95% CI]	8 (27.6) [11.3–43.9]	0 (0.0) [0.0–0.0]	5 (7.9) [1.2–14.6]	0.021
Intra-abdominal Abscess, n (%) [95% CI]	4 (13.8) [1.3–26.3]	1 (7.7) [0.0–22.2]	5 (7.9) [1.2–14.6]	0.456
Auto-amputation, n (%) [95% CI]	3 (10.3) [0.0–21.4]	2 (15.4) [0.0–35.0]	7 (11.1) [3.3–18.9]	1.00
Gangrene, n (%) [95% CI]	7 (24.1) [8.5–39.7]	2 (15.4) [0.0–35.0]	0 (0.0) [0.0–0.0]	<0.001
Hematoma, n (%) [95% CI]	1 (3.4) [0.0–10.0]	0 (0.0) [0.0–0.0]	4 (6.3) [0.3–12.3]	1.00

## Data Availability

The raw data supporting the conclusions of this article will be made available by the authors on request.

## Abbreviations

The following abbreviations are used in this manuscript:

CT	Computed Tomography
PTA	Post-traumatic Appendicitis
AIR	Appendicitis Inflammatory Response
PAS	Pediatric Appendicitis Score

## Appendix A

**Table A1:** Comprehensive clinical and demographic overview of the 106 historical cases of post-traumatic appendicitis (PTA) included in the quantitative analysis, spanning a century of literature. Cases are categorized into pediatric (≤18 years) and adult (>18 years) cohorts. For studies reporting case series, age is presented as the mean value with the corresponding range in parentheses.

Paper	Year	Cases (n)	Age (Years)	Sex	Cause of Trauma	Diagnosis	Appendicolith	Complications
Pediatric Cases								
Ramsook C [2]	2001	1	12	M	kick in the abdomen	CT scan	yes	no
Pisoni et al. [12]	2006	1	10	F	blunt abdominal trauma		no	periappendicitis
Salinas-Castro et al. [13]	2022	1	14	F	soccer ball trauma	CT scan	no	no
Habachi et al. [14]	2023	1	12	M	fall	US	no	perforation, abscess
Etensel et al. [16]	2005	5	10 (5–14)	M (5)	MVC (4), fall/polytrauma (1)	surgery (5)	no (5/5)	perforation/pneumoperitoneum (1)
Taylor CJ [18]	1923	1	18	M	hit by an elbow while playing football	surgery	no	no
Bissell et al. [19]	1928	3	14 (10–18)	M (3)	fall (2), kick (1)	surgery (3)	yes (3/3)	perforation (2), abscess (1), peritonitis (2)
Marcus et al. [20]	1931	5	11 (9–14)	M (4), F (1)	kick (1), MVC (1), fall (3)	surgery (5)	yes (1/5)	perforation (2), peritonitis (1), gangrenous (1)
Shutkin et al. [21]	1936	1	14, N/A	M (2)	kick (1), fall (1)	surgery (2)	yes (1/2)	gangrenous (2), perforation (2)
Rhodes et al. [22]	1940	3	11 (11–13)	M (1), F (2)	hit by a volley ball (1), hit by a baseball (1), fall (1)	surgery (3)	no (3/3)	gangrenous (1), perforation (2), hemorrhageshemorragies (1)
Bhajekar et al. [23]	1942	1	13	M	fall	surgery	no	abscess
Black WR [24]	1948	2	14, 14	M (1), F (1)	bicycle fall (1), fall (1)	surgery (2)	yes (2/2)	abscess, gangrenous (2)
Hennington et al. [25]	1991	1	12	M	bicycle fall	surgery	no	no
Musemeche et al. [26]	1995	1	4	M	road car accident	CT scan	no	
Ciftci et al. [27]	1996	3	11 (7–14)	M (2), F (1)	hit by a ball (1), MVC (1), assault (1)	surgery (1), CT scan (2)	no (3/3)	perforation (3)
Serour et al. [28]	1996	3	8 (7–11)	M (3)	punch (1), fall (1), fight (1)	CT scan (1), surgery (2)	yes (1/3)	gangrenous/periappendicitis (1), perforation/abscess (1)
Osterhoudt KC [29]	2000	1	9	M	hit by a car	US	no	perforation
Paul G [30]	2000	1	16	M	seatbelt collision	surgery	no	perforation, auto-amputation
Houry et al. [31]	2001	1	5	M	fall	CT scan	no	perforation
Ramesh et al. [32]	2002	1	11	M	bicycle fall	US	no	perforation, appendicitis
Sumer et al. [33]	2008	1	8	M	fall			perforation
Amir et al. [34]	2009	1	10	M	trauma from a desk’s corner	CT scan	no	no
Toumi et al. [35]	2010	1	11	M	hit by an elbow	CT scan	no	peritonitis, abscess
Torres-Grau et al. [36]	2012	1	15	M	bicycle fall	surgery	no	non
Paschos et al. [37]	2012	1	17	F	bicycle fall	US		no
Wani I [38]	2013	4	13 (9–17)	M (2), F (2)	bicycle fall (1), kick in the abdomen (2), fall (1)	US/CT scan (4)	yes (2/4)	mesoappendicular hematoma (1)
Reyes-Hernández et al. [39]	2013	1	14	F	bullying	US		no
Goldman et al. [40]	2013	1	11	M	punch in the abdomen	MRI	no	no
Moslemi et al. [41]	2013	1	13	M	bicycle fall	CT scan	no	auto-amputation, pneumoperitoneum
Ahmed et al. [42]	2014	1	12	M	trauma from a desk’s corner	US	no	perforation, shock, hemoperitoneum, peritonitis
Jensen et al. [43]	2016	1	7	M	bicycle fall	CT scan	yes	auto-amputation
Cobb [44]	2017	1	17	M	MVC	CT scan	no	peritonitis
Klis et al. [45]	2017	1	12	F	bicycle fall	US	no	perforation
Singh et al. [46]	2018	1	10	M	bicycle fall	surgery	no	no
Çağlar [47]	2018	1	12	M	fall from a swing	RX/CT scan	no	no
Zvizdic et al. [48]	2020	1	7	M	hors hoof kick	CT scan	no	perforation, peritonitis
Sharma et al. [49]	2021	1	11	M	bicycle fall	CT scan	no	perforation, pneumoperitoneum, appendiceal auto-amputation
Chalh et al. [50]	2021	1	8	M	fall on a boulder	US	yes	nno
Kerkeni et al. [51]	2022	1	8	M	bicycle fall	surgery	no	auto-amputation, perforation, pneumoperitoneum
Albalawi et al. [52]	2023	1	10	M	Fall fall from a trampoline	CT scan	no	no
Jaramillo et al. [53]	2024	1	18	F	MVC	CT scan	no	no
DuBois et al. [54]	2025	1	13	F	MVC	CT scan	yes	no
Namdeo S [55]	2025	1	11	M	hit by a football	surgery	no	no
Gupta et al. [56]	2016	2	9, 11	M (1), F (1)	bicycle fall (1), fall on a boulder (1)	US (1), surgery (1)	yes (2/2)	perforation with abscess (1)
Adult Population								
Alabdulaaly et al. [15]	2024	1	21	F	Road road traffic accident	CT scan	no	perforation
Behan RJ [57]	1927	1	28	M	fall on a tomato patch	surgery	no	perforation, peritonitis
Bissell et al. [19]	1928	1	19	M	hit on the abdomen during a fight	surgery	yes	perforation, peritonitis
Marcus et al. [20]	1931	1	47	M	hit by a carbon brick	surgery	no	peritonitis
Maes et al. [58]	1935	1	27	M	calf kick	surgery	no	haemorragies
Rhodes et al. [22]	1940	4	27 (21–37)	M (4)	fall (2), hit by a timber (1), hit by a knee (1)	surgery (4)	yes (1/4)	perforation (4), peritonitis (2), abscess (1)
Gatewood et al. [59]	1956	1	39	F	MVC	surgery		auto-amputation
Fiorini et al. [60]	1974	1	21	M	MVC	surgery	no	perforation
Geer et al. [61]	1975	1	39	M	MVC	surgery	no	auto-amputation, appendiceal infarct
Thomas DF [62]	1978	1	36	M	MVC	surgery	yes	auto-amputation
Hennington et al. [25]	1991	1	46	M	trauma due to car fall	surgery	no	gangrenosa
Bangs RG [63]	1991	1	20	M	MVC	surgery	no	no
Stephenson et al. [64]	1995	1	32	F	seat belt collision	surgery	no	no
Edwards et al. [65]	1999	1	41	M	MVC	CT scan	no	auto-amputation
Takagi et al. [66]	2000	1	45	F	seat belt collision	surgery	no	periappendicitis
Tekin et al. [67]	2001	1	36	M	punch and hit	surgery	no	perforation, hemorragieshemorrhages
Hagger et al. [68]	2002	1	60	M	fall from a ladder	CT scan	no	abscess
Karavokyros et al. [69]	2004	1	21	M	robbed and hit	surgery	no	no
Derr et al. [70]	2009	1	41	M	fall while cliff diving	US	no	no
Atalla et al. [71]	2010	1	53	M	fall on a car door	CT scan	no	no
Faulkner et al. [72]	2010	1	28	F	MVC	CT scan	no	appendicular-cutaneous fistula
O’Kelly et al. [73]	2012	1	40	M	blunt abdominal trauma	surgery	no	perforation
Wani I [38]	2013	4	49 (34–64)	M (2), F (2)	fall (3), kick (1)	US/CT scan (4)	yes (3/4)	appendicular lump (1), perforation (1), mesoappendicular haematoma (1)
Altunkaş et al. [74]	2015	1	32	F	fall	US	yes	caecal hematomas
Ross et al. [75]	2016	1	59	F	MVC	CT scan	yes	no
Go et al. [76]	2016	1	23	M	seat belt collision	surgery	no	auto-amputation
Latorre et al. [77]	2017	1	26	M	MVC	surgery	no	perforation, peritonitis
Khilji et al. [78]	2017	1	43	M	MVC	CT scan	no	no
AlJaberi et al. [79]	2018	1	24	M	seat belt collision	CT scan	no	auto-amputation
Siddiqui J [80]	2018	1	22	M	Fall from a ladder (3 m)	CT scan	no	no
Toffaha et al. [81]	2021	1	35	M	camel kick	CT scan	no	no
Alharmoodi et al. [82]	2022	1	53	M	MVC	CT scan	no	hemoperitoneum, auto-amputation
Demir et al. [83]	2024	1	65	M	seat belt collision	CT scan	no	periappendicitis
Shawasha et al. [84]	2024	1	28	M	hit by a knee (fall)	CT scan	no	perforation, abscess
Karapolat et al. [85]	2018	1	36	M	bicycle fall	US/CT scan	no	no
Our Case	2026	1	10	M	hit by a hockey stick	US	no	no
